# Developing a highly efficient and wildly adaptive CRISPR‐*Sa*Cas9 toolset for plant genome editing

**DOI:** 10.1111/pbi.13047

**Published:** 2019-02-12

**Authors:** Ruiying Qin, Juan Li, Hao Li, Yuandi Zhang, Xiaoshuang Liu, Yuxin Miao, Xiuqing Zhang, Pengcheng Wei

**Affiliations:** ^1^ College of Food Science and Nutritional Engineering China Agricultural University Beijing China; ^2^ Key Laboratory of Rice Genetic Breeding of Anhui Province Rice Research Institute Anhui Academy of Agricultural Sciences Hefei China

**Keywords:** *Sa*Cas9, PAM, gene editing, base editor, rice

## Accession numbers

The accession numbers of the targeted genes by the SaCas9 toolset in this study are: *PDS*:* Os03g0184000*;* DL*:* Os03g0215200*;* NAL1*:* Os04g0615000*;* SLR1*:* Os03g0707600*;* IPA1*:* Os08g0509600*;* TAC1*:* Os09g0529300*;* Ehd1*:* Os10g0463400*;* Pi‐d2*:* Os06g0494100*;* OsMKK6*:* Os01g051010 0*;* OsMPK3*:* Os03g0285800*;* Pi‐ta*:* Os12g0281300*;* OsSPL17*:* Os09g0491532*;* GL2*:* Os02g0701300*;* OsGRF3*:* Os04g0600900*;* Wx*:* Os06g0133000*.

The next‐generation sequencing data of mutation genotyping could be achieved at NCBI BioProject ID PRJNA496604.

## Conflict of interest

The authors declare no conflict of interest.

Dear Editor,

The CRISPR‐*Streptococcus pyogenes* Cas9 (*Sp*Cas9) system offers a rapid, simple and flexible genome editing approach. However, the targeting scope of the *Sp*Cas9 system is limited by the canonical NGG PAM. To broaden the editing range of the CRISPR system, several Cas orthologs recognized different PAM were isolated from diverse microbes and were engineered as powerful genome editing tools in eukaryotic cells (Murovec *et al*., 2017). One Cas9 ortholog, *Staphylococcus aureus* Cas9 (*Sa*Cas9), has a smaller size and comparable activity compared to *Sp*Cas9 (Kleinstiver *et al*., 2015a,b). Previous studies reported that the *Sa*Cas9 could induce highly efficient targeted mutagenesis in Arabidopsis, citrus, tobacco and rice (Murovec *et al*., 2017). *Sa*Cas9 recognizes a longer PAM motif (NNGRRT) than *Sp*Cas9 (Kleinstiver *et al*., 2015b). In mammalian cells, the PAM specificity of *Sa*Cas9 could be relaxed to NNNRRT by a KKH variant (*Sa*Cas9‐KKH, *Sa*KKH; Kleinstiver *et al*., 2015a). In this study, we investigated the targeting capability of *Sa*KKH in rice. Furthermore, we developed cytosine base editors (CBEs) and adenine base editors (ABEs) based on *Sa*Cas9 and *Sa*KKH.

**Figure 1:**
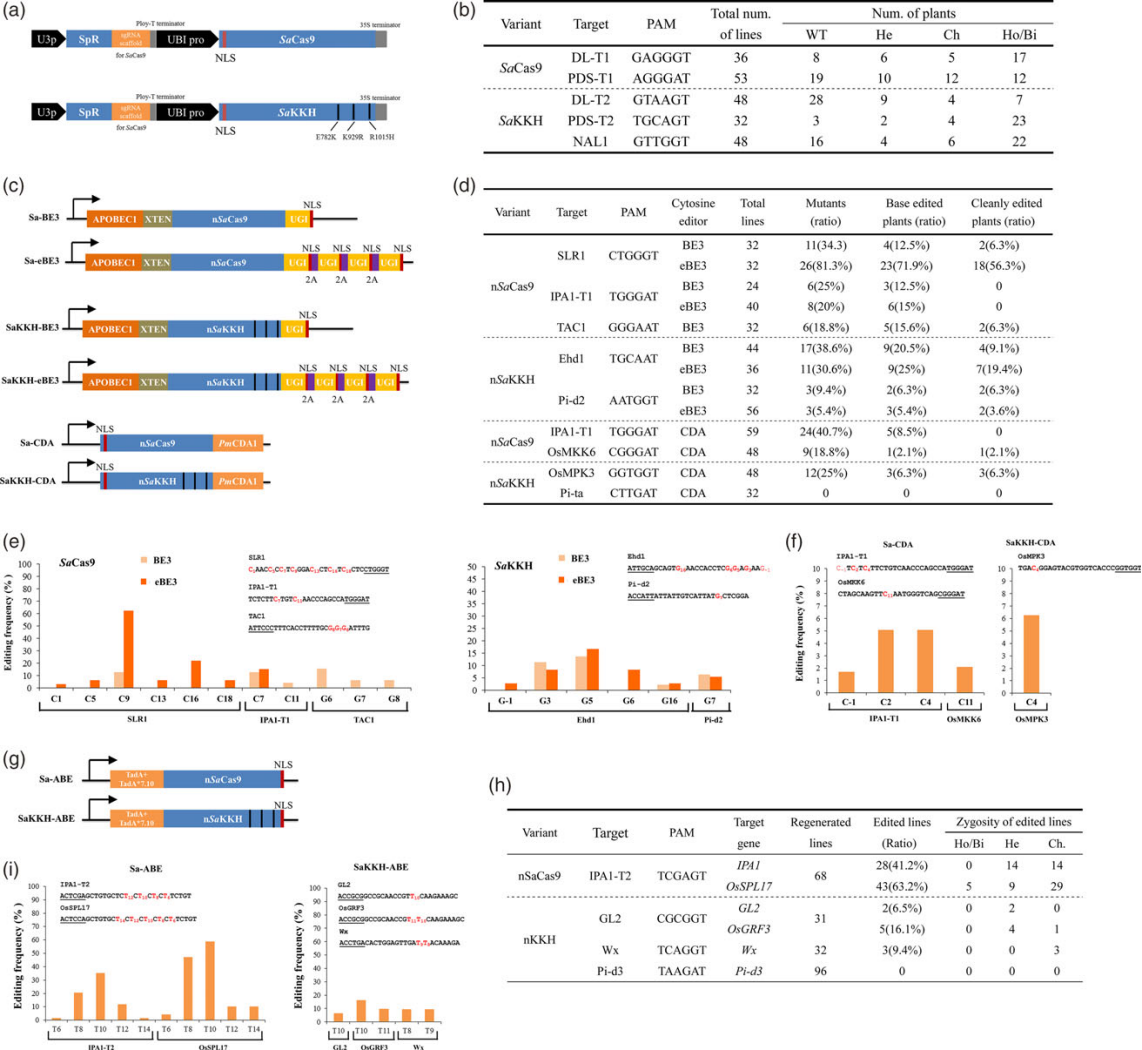
Rice genome editing generated by *Sa*Cas9 toolset. (a) Schematic illustration of the sgRNA and Cas9 expression cassettes of plant SaCas9 systems. To express sgRNA, a 21 bp protospacer sequence was inserted downstream of the rice U3 promoter (U3p) to replace the spectinomycin resistance gene (SpR). A maize ubiquitin promoter (UBI pro) was used to express *Sa*Cas9 or *Sa* KKH. (b) Mutations induced by *Sa*Cas9 and *Sa* KKH in regenerated rice plants analysed by Hi‐TOM assay with a 5% threshold (http://www.hi-tom.net/hi-tom). WT, wild‐type sequence in the target region; He, heterozygous mutation; Ch, chimeric mutation; Ho/Bi, homozygous or biallelic mutation. (c) Schematic illustration of *Sa*Cas9‐CBEs. (d) Cytosine editing induced by *Sa*Cas9 base editors in regenerated rice plants. The regenerated plants with exclusive C‐to‐T base conversions were considered as cleanly edited plants. (e) Frequencies of the base editing induced by *Sa*Cas9‐BE3 base editors at different C(G)s in the target sequence. The PAM sequence is underlined, and the targeted bases and positions in the protospacer are labeled in red. Sa‐eBE3 was not tested at the TAC1 target. (f) Frequencies of the base conversions induced by *Sa*Cas9‐CDA base editors at different C(G)s in the target sequence in regenerated plant populations. (g) Schematic illustration of *Sa*Cas9‐ABE base editors. (h) Adenine editing induced by *Sa*Cas9 base editors in regenerated rice plants. (i) Frequencies of the A‐to‐G conversion induced by *Sa*Cas9 ABEs at different A(T)s in the target sequence.

To achieve a high efficiency in monocots, we re‐optimized the codons of *Sa*Cas9 for rice. To broaden the targeting range of *Sa*Cas9, three key mutations (E782K/N968K/R105H) were simultaneously introduced to generate *Sa*KKH (Figure 1a). The activity of *Sa*Cas9 was identified in *PDS* and *DL* genes *via* stably *Agrobacterium*‐mediated transformation of *Japonica* rice. The targeted mutations were determined in regenerated plants by high‐throughput tracking of mutation (Hi‐TOM) detection (Liu *et al*., 2018). After generating transgenic plants, 34 out of 53 lines and 28 out of 36 lines carried targeted mutations in the PDS‐T1 and DL‐T1 regions, achieving 64.2% and 77.8% mutagenesis efficiency respectively. This result confirms that *Sa*Cas9 efficiently edits crop genome. To examine the genome editing activity of *Sa*KKH in plants, three protospacers with a NNNRRT PAM were further designed in rice *PDS*,* DL* and *NAL1* genes. At the PDS‐T2 target with a TGCAGT PAM, mutations were detected in 90.6% of *Sa*KKH lines (Figure 1b). In addition, we found that most of the mutated lines were homozygous or biallelic mutants (79.3%), further indicating the high‐mutagenesis frequency induced by *Sa*KKH. Moreover, targeted mutations were obtained at the DL‐T2 and NAL1 sites at rates of 41.7% and 66.7% respectively.

A series of CBE tools was recently developed by fusing cytosine deaminase to *Sp*Cas9 nickase (n*Sp*Cas9; Komor *et al*., 2016; Nishida *et al*., 2016). At first, we constructed *Sa*Cas9 CBEs by attaching the optimized sequence of rat *APOBEC1* to the 5′ terminus and the optimized sequence of *uracil glycosylase inhibitor* (*UGI*) to the 3′ terminus of *nSaCas9 (D10A)* and *nSaKKH(D10A) nickase*, leading to Sa‐BE3 and SaKKH‐BE3 respectively. To increase the editing product purity, we separately fused three copies of UGI to the C terminus of Sa‐BE3 and SaKKH‐BE3, generating Sa‐eBE3 and SaKKH‐eBE3 (Figure 1c). A protospacer was designed to edit the C290 in *SLR1* gene, using Sa‐BE3 or Sa‐eBE3. We found 11 out 40 Sa‐BE3 lines had mutations in the target region. Among them, 4 lines (10% frequency) carried base conversions, which were located at position 9 of the protospacer (the nucleotide at the 5′ end is position 1; Figure 1d,e). The ratio of base editing in *SLR1* induced by Sa‐eBE3 was much higher (Fisher's exact test, *P* < 0.05). We found 81.3% of the regenerated lines had targeted mutations, and 71.9% of the lines were edited. More than half of the lines had only clean C‐to‐T conversion(s) in the target region (designated as clean editing). In addition, we found homozygous or biallelic clean base editing in 10 of 32 lines, further indicating the extraordinary activity of Sa‐eBE3 in this target. The base conversion at position 9 has highest frequency, while the editing also occurred at positions 1, 5, 9, 13, 16 and 18 (Figure 1e), suggesting the Sa‐eBE3 have a broad editing window. The miR156 binding region in the *IPA1* gene was also used to design a sgRNA (IPA‐T1). Screening of 24 Sa‐BE3 lines and 40 Sa‐eBE3 lines detected 2 and 6 edited lines respectively. Moreover, the 3′ splicing point of the 4th intron of rice *TAC1* was targeted by Sa‐BE3. The base editing of G (s) was obtained in 15.6% regenerated plants. To test the editing activity of *Sa*KKH BE3s, the protospacer with TGCAAT and AATGGT was designed to target the G655 and G1383 in *Ehd1* and *Pi‐d2* genes respectively. We found that 20.5% and 6.3% of SaKKH‐BE3 plants have targeted base conversions in the Ehd1 and Pi‐d2 target region respectively (Figure 1D). Furthermore, we noticed that the editing efficiency at the Ehd1 target was increased to 25% by the SaKKH‐eBE3 vector. In addition, the clean editing frequency induced by SaKKH‐eBE3 is increased to 19.4% from the 9.1% generated by SaKKH‐BE3. The SaKKH‐eBE3‐induced base conversion ratio remained as low as 5.4% at the Pi‐d2 target, which may be caused by the inconvenient GC context for the rat APOBEC1 activity. The base conversions in the Ehd1 target were occurred in Gs at position 3, 5, 6, 16 in the 21 bp target sequence and the G immediately 5′ upstream of the protospacer (position ‐1), while the editing on the Pi‐d2 target were only detected in the G at position 7 (Figure 1e). Very recently, a similar SaKKH‐BE3 was constructed and assembled into the pRCBEsakkh‐OsU6sa vector (CBE‐P5; Hua *et al*., 2018b). The editing activity of CBE‐P5 was not observed at the target in the rice PMS1 gene (Hua *et al*., 2018b), suggesting insufficient activity of SaKKH‐BE3. However, the mutagenesis efficiency introduced by the SaKKH‐BE3 in this study reached as high as 38.6%. Because the activity of CBE‐P5 was examined at only one site (Hua *et al*., 2018b), the editing efficiency of the two systems cannot be directly compared based on current results.

To expand the editing scope of the *Petromyzon marinus* cytidine deaminase 1 (*Pm*CDA1) ‐based CBE in plant (Shimatani *et al*., 2017), the n*Sa*Cas9 or n*Sa*KKH nickase was separately assembled with a *Pm*CDA1 domain, which was designated Sa‐CDA or SaKKH‐CDA respectively (Figure 1c). Two targets were selected and examined for each CDA base editor. For Sa‐CDA, the IPA‐T1 protospacer of Sa‐BE3 was used. In 59 regenerated plants, base editing was observed in 5 lines (Figure 1d). The substitutions occurred at the position 2 and 4 of the protospacer and the position ‐1 out of the protospacer (Figure 1f). At another target site in *OsMKK6*, one cleanly edited line out of 48 screened regenerated plants was obtained. For SaKKH‐CDA, protospacers were designed to edit the TEY domain of *OsMPK3* and the C2753 of *Pi‐ta*. At the OsMPK3 target, 3 out 48 lines carried a clean C‐to‐T conversion, all of which were located at position 4; while no mutation was detected in the *Pi‐ta* target with a CTTGAT PAM.

Adenine base editors convert A:T to G:C in the target site (Gaudelli *et al*., 2017). To increase the range of ABEs in plants, a rice codon‐optimized TadA‐XTEN‐TadA*7.10 fragment was assembled into the n*Sa*Cas9 (D10A) or n*Sa*KKH (D10A) nickase, resulting Sa‐ABE and SaKKH‐ABE, respectively (Figure 1g). A protospacer (IPA‐T2) that simultaneously targeted two genomic sites in *IPA1* and *OsSPL17* was selected for testing Sa‐ABE. The editing occurred in *IPA1* and *OsSPL17* with 41.2% and 63.2% efficiency respectively (Figure 1h). In both targets, the Cs at positions 8 and 10 were edited with higher frequency (Figure 1i). Other than the targeted base conversions, an undesired mutation (a 35 nt deletion) was found in only one plant at the OsSPL17 site, indicating high‐editing purity of Sa‐ABE. Moreover, 21 lines (31.9%) were edited at both sites, suggesting the capability of multiplexed editing with Sa‐ABE. This result is similar to the editing generated by the ABE‐P2 in a recent report (Hua *et al*., 2018a). We notice that the SaKKH‐ABE (ABE‐P5) was also recently reported with relatively lower (0%–6.5%) efficiency compared to Sa‐ABE in plant (Hua *et al*., 2018b). We further examined the activity of the SaKKH‐ABE with three independent protospacers with NNHRRT PAMs. For the protospacers targeting the *Wx* and *Pi‐d3* gene, the targeting efficiency was 0% and 9.4% respectively. In addition, The GL2 protospacer simultaneously targeted *GL2* and *OsGRF3* genes. We found that only 6.5% of plants (2 out of 31 lines) carried A‐to‐G conversions in the *GL2* gene, while the editing frequency reached 16.1% (five edited lines) in *OsGRF3*, suggesting the SaKKH‐ABE may have potential to achieve efficient editing in some targets.

In this study, we developed a series of mutagenesis and base editing tools using *Sa*Cas9 and its derivative for plant genome editing. These tools expand the scope of genome editing to targets with a NNNRRT PAM. It is desired to simultaneously perform different editing events, such as base editing and targeted mutation, in a single plant. Using the *Sa*Cas9 tools, this purpose would be robustly achieved by co‐transforming or stacking the previously established *Sp*Cas9 systems. Taking these results together, we established a highly efficient and wildly adaptive *Sa*Cas9 toolset that may advance plant research and accelerate crop improvement.
